# An unusual case of a tension pneumothorax

**DOI:** 10.1093/jscr/rjac496

**Published:** 2022-11-16

**Authors:** Bassam Redwan, Robert Kirstein, Volkan Kösek, Burkhard Thiel, Hubert Zirngibl, Björn Schmitz

**Affiliations:** Department of Thoracic Surgery, Klinik am Park, Klinikum Westfalen, Lünen, Germany; Department of General Surgery, Klinik am Park, Klinikum Westfalen, Lünen, Germany; Department of Thoracic Surgery, Klinik am Park, Klinikum Westfalen, Lünen, Germany; Department of Thoracic Surgery, Klinik am Park, Klinikum Westfalen, Lünen, Germany; Depertment of Surgery, Helios University Hospital Wuppertal, University of Witten/Herdecke, Wuppertal, Germany; Department of General Surgery, Klinik am Park, Klinikum Westfalen, Lünen, Germany

## Abstract

A total intra-thoracic stomach describes the case of a complete herniation of the stomach into the thoracic cavity. Symptoms may vary from mild to an acute life-threatening situation in case of perforation or bleeding, requiring emergency surgery. Here we describe the case of a gastric perforation leading to a tension pneumothorax and concomitant pleural empyema due to a giant hiatal recurrence after previous surgery. Multidisciplinary management involving thoracic surgeons helped in achieving the best clinical outcome for the patient.

## INTRODUCTION

A total intra-thoracic stomach describes the case of a complete herniation of the stomach into the thoracic cavity. Symptoms may vary from mild to an acute life-threatening situation in case of perforation or bleeding, requiring emergency surgery. Here we describe the case of an unusual complication after surgery for a hiatal hernia.

## CASE PRESENTATION

**Figure 1:**
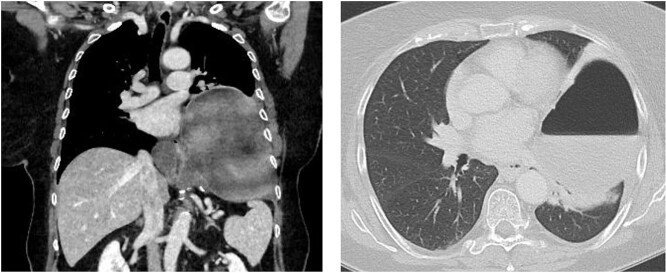
Chest/abdomen computed tomography showing a new recurrence with complete up-side-down stomach and compression of the left lower lung lobe.

A 67-year-old female patient was admitted to the emergency department of our hospital with upper abdominal pain and vomiting. Previous medical history included a laparoscopic hiatal hernia repair in October 2018 due to a type III hiatal hernia and re-do surgery with mesh repair and a Toupet 270° posterior wrap due to a recurrence of the hiatus hernia with gastroesophageal reflux disease (GERD) in May 2019. Imaging studies revealed a new recurrence with total intra-thoracic stomach and compression of the left lower lung lobe (Fig. 1).

**Figure 2:**
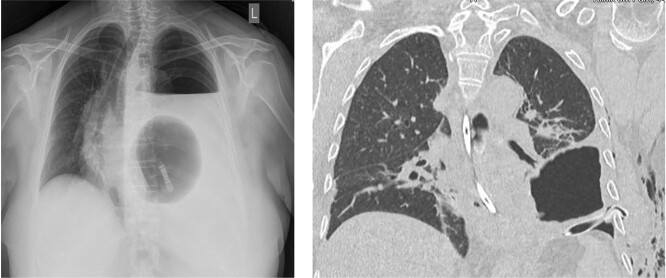
Chest X-ray showing a large left-sided tension seropneumothorax with a right-sided mediastinal shift. Chest CT-scan after insertion of a chest tube.

**Figure 3:**
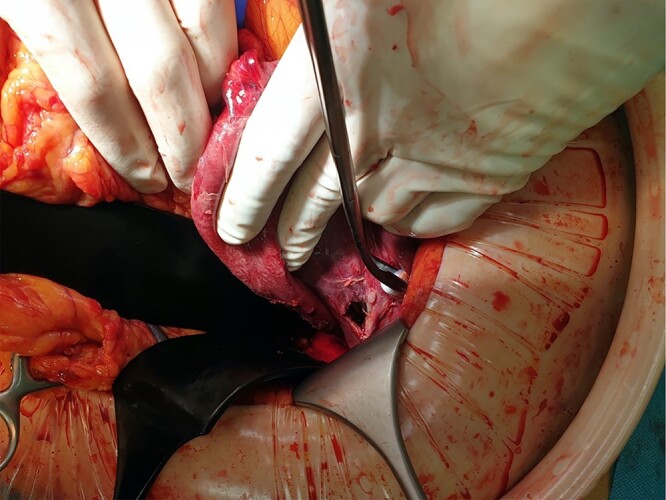
Intraoperative finding with the gastric perforation.

One day after admission, the patient developed massive dyspnea with a rapid deterioration of general condition. Chest X-ray showed a large left-sided tension seropneumothorax with a right-sided mediastinal shift (Fig. 2). Uncomplicated, ultrasound-guided chest drain insertion was performed immediately, and the clinical condition improved instantly. However, gastric secretion through the chest tube was observed and gastric perforation as a cause for the seropneumothorax was suspected. Therefore, emergency surgery was performed. A lift-sided antero-lateral thoracotomy was performed to rule out any lung parenchym lesions. Intraoperatively, a 2 × 2 cm gastric perforation was found (Fig. 3). After excision, the perforation was sutured, and the stomach was transposed into the intraabdominal cavity. Fibro-purulent empyema (stage II) was observed and local pleurectomy and decortication of the left lower lobe were performed. A transverse upper laparotomy was performed. After adhesiolysis due to massive adhesions following previous surgeries, hernia sac was excised, mobilization of the esophagus was carried out and crura were closed. Due to the present infection, no alloplastic material was used. A partial Watson anterior wrap was performed. There were no intraoperative complications. The patient was extubated in the operation room and transferred to the intermediate care unit for further observation. The postoperative course was uneventful, and patient was discharged in good condition on POD 7. Histological examination revealed a Helicobacter-positive gastric ulcer as a cause for gastric perforation. Two months after discharge, gastroscopy was performed and showed a normal finding with no signs of recurrence of the hiatal hernia.

## DISCUSSION

Intrathoracic perforation of the stomach in case of giant hiatal hernia is a rare complication and has been only described in a few case reports [1–4]. However, tension pneumothorax was not present in any of these cases. Prevalence and incidence of hiatal hernia vary from 10 to 80% due to the different diagnosis criteria and the asymptomatic patients [5]. Hiatal hernia is divided into four groups depending on the anatomy. These include sliding axial (type I), paraesophageal (type II), mixed (type III) or combined (type IV), where intrathoracic herniation of abdominal viscera other than the stomach through the diaphragmatic hiatus is present.

In the present case, recurrence of a giant paraesophageal hernia with herniation of the complete stomach into the left intrathoracic cavity was observed. Such hernias resemble 5–15% of all hiatal hernias [6].

In type I hernias, surgery is only performed in symptomatic patients for treatment of GERD, when medical therapy has failed to achieve clinical improvement. Surgical hernia repair is indicated in types II–IV of hiatal hernias [7].

Pneumothorax after hiatal surgery may occur in the early postoperative period, when the pleura is accidentally injured during surgery. In such cases, pneumothorax is early detected by chest X-ray and treated by insertion of a chest tube [8].

In our case, perforation of the herniated stomach caused a tension pneumothorax requiring immediate insertion of a chest tube. Histological examination revealed presence of a *Helicobacter pylori*-positive ulcer. Whether ulcera a more common in such cases is unknown but may be a result of the longer exposition of the intrathoracic herniated stomach to gastric secretion. A further reason for perforation may be ischemic complications of the stomach wall in case of chronic incarceration. In rare cases, occult so known Cameron ulcera may occur. Furthermore, ischaemia of the stomach due to volvulus within the chest may result in perforation and this may require resectional surgery.

Conservative treatment of giant hiatal hernia is also reported by several studies. Oude Nijhuis *et al*. reported long-term results after conservative treatment of giant paraesophageal hernia [9]. Of *n* = 186 conservativly treated patients, elective surgery was necesarry in *n* = 13 patients due to symptom progression and in *n* = 2 patients, emergency surgery for hernia-related compliactions was performed. Hence, conservative therapy for asymptomatic patients with giant hernias seems to be a safe treatment concept [9]. The challenge is early detection of symptom progression and possible complications to decrease the rate of emergency surgery in this setting.

The number of detected giant hiatal hernias increases in the elderly population. One reason is that this patient-cohort undergoes more imaging diagnostic studies, in which more asymptomatic hernias are diagnosed [10]. Due to the higher rate of comorbidity in the elderly, surgery is associated with a higher perioperative risk. Therefore, surgery should only be considered in well-selected patients or in case of life-threatening hernia-related complications.

When surgery is indicated, unless the patient underwent previous upper abdominal or left-sided thoracic surgical procedures, the laparoscopic approach should be elected, since it is safe with a low rate of recurrence and high patient comfort [11].

In conclusion, gastric perforation leading to a tension pneumothorax in case of giant hiatal hernias is a rare complication and may lead to a life-threating situation. In case of previous surgeries, an open surgical approach may offer a better exposure of the surgical site. When concomitant pleural empyema is present, multidisciplinary management involving thoracic surgeons helps in achieving the best clinical outcome.
